# Alpha-fetoprotein-producing primary lung carcinoma: A case report

**DOI:** 10.1186/1477-7819-9-47

**Published:** 2011-05-09

**Authors:** Masahiro Kitada, Keisuke Ozawa, Kazuhiro Sato, Yoshinari Matsuda, Satoshi Hayashi, Yoshihiko Tokusashi, Naoyuki Miyokawa, Tadahiro Sasajima

**Affiliations:** 1 Department of Surgery, Asahikawa Medical University, Japan; 2 Department of Clinical Pathology, Asahikawa Medical University. Japan

## Abstract

Alpha-fetoprotein (AFP)-producing lung adenocarcinoma is a rare type of lung cancer, with its characteristics not yet fully clarified. We recently encountered a case of this type of lung cancer. The patient was a 69-year-old man who consulted an internist with the chief complaint of epigastric pain. Chest X-ray and CT revealed a lobulated mass measuring 70 mm in diameter in the right lower lung field and a metastasis in the right hilar lymph nodes. Of the tumor markers, the serum AFP was elevated (4620 ng/ml), and the serum carcinoembryonic antigen and carbohydrate antigen 19-9 were also slightly elevated. Transbronchial lung biopsy revealed the diagnosis of lung cancer. Under thoracoscopic assistance, right lower lobectomy + mediastinal lymph node dissection was carried out. Immunostaining showed the tumor cells to be AFP-positive. The tumor was thus diagnosed as an AFP-producing lung adenocarcinoma. The patient followed an uneventful clinical course after the surgery, with serum AFP decreasing to the normal range by about 2 weeks after the surgery. As of this writing, no sign of tumor recurrence has been noted. This case is presented here with a review of the literature.

## Background

Aalpha-fetoprotein (AFP) is a type of protein formed in the fetal liver and yolk sac and is detected in the fetal serum. In regard to the pathological significance of this protein in adults, serum AFP is often elevated in patients with liver cancer or gonadal germ cell tumors, such as yolk sac tumor. Because the serum AFP level decreases in response to effective treatment, measurement of the serum AFP level is carried out during follow-up of patients after treatment or for the detection of tumor recurrence. AFP-producing ovarian cancer and gastric cancer have also been reported, whereas AFP-producing liver cancer is rare. Because AFP-producing lung cancer has scarcely been reported, the clinical features of this type of lung cancer are still unclear. In this context, we report a case of this type of cancer that we encountered recently.

## Case presentation

**Figure 1:**
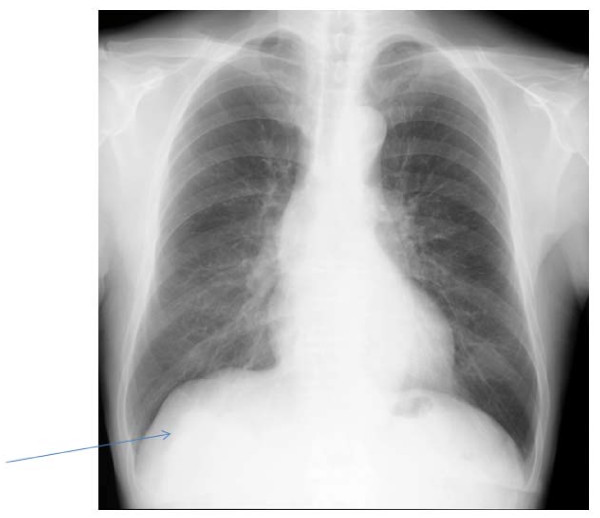
**A chest x-ray revealed a mass measuring 65 mm in diameter in the right lower lung field**.

**Figure 2:**
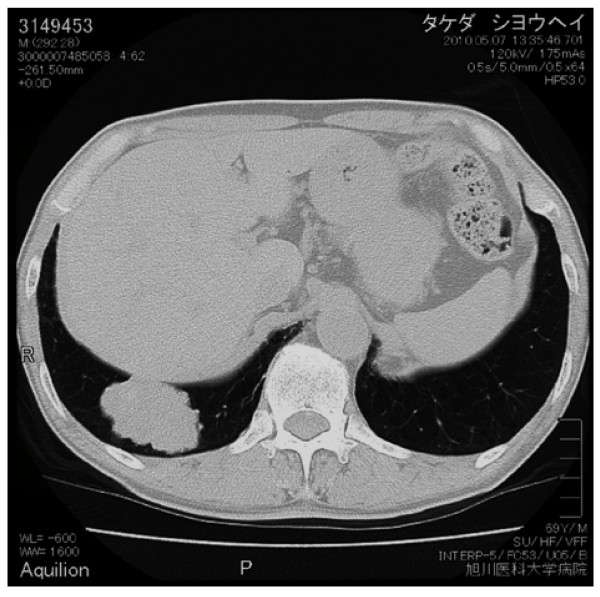
Chest CT revealed a lobulated mass measuring 65 mm in diameter involving S9 and S10 of the right lung

**Figure 3:**
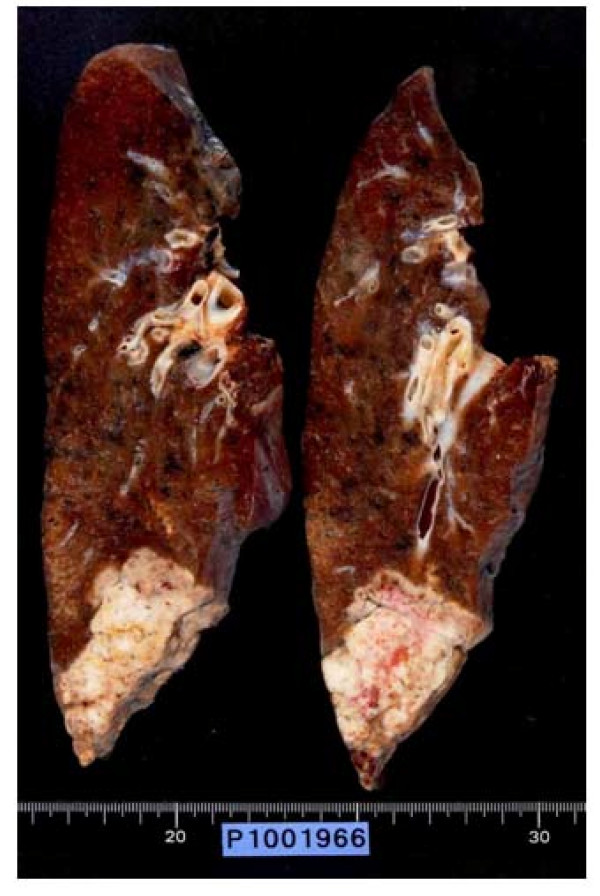
A macroscopic specimen showed that the resected tumor measured 6.5 cm in diameter and was a solid tumor

**Figure 4:**
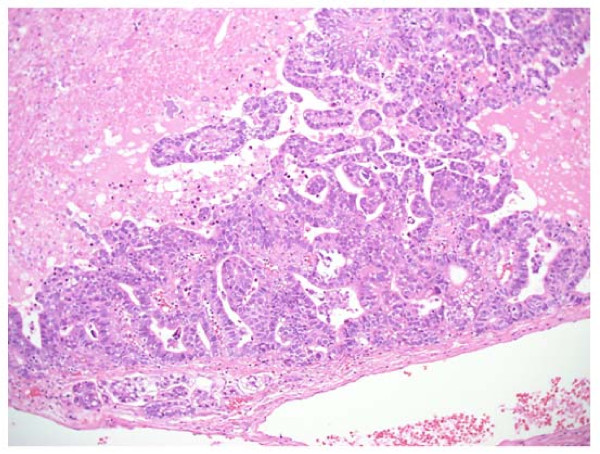
Histological findings of tumor showed pulmonary adenocarcinoma (HE × 100)

**Figure 5:**
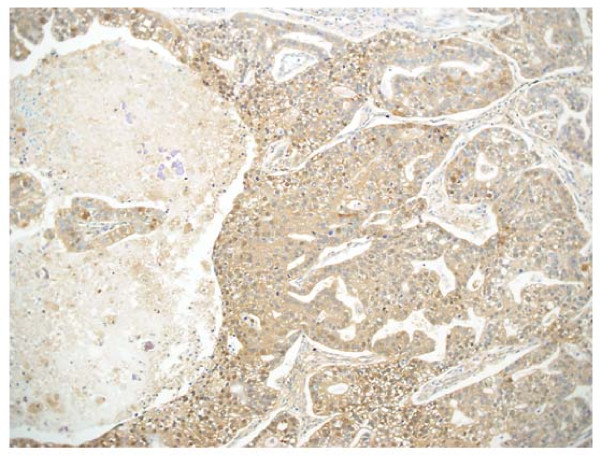
Immnohistochemical findings showed that positive staining of a number of tumor cells for AFP, leading to the diagnosis of AFP-producing lung adenocarcinoma. (×100)

A 69-year-old man consulted a nearby internal medicine clinic with the chief complaint of epigastric pain. He was diagnosed as having gastroesophageal reflux and initiated on treatment. A chest x-ray performed at that time revealed a mass in the right lower lung field. The patient had a history of smoking (Brinkman index: 1800) and had been diagnosed earlier as having pulmonary emphysema. He had the past of the alcoholic hepatitis. His family history was not noteworthy. He was 160 cm tall and weighed 55 kg. Physical examination revealed no abnormalities. A chest x-ray revealed a mass measuring 65 mm in diameter in the right lower lung field (Figure 1). Chest CT revealed a lobulated mass measuring 65 mm in diameter involving S9 and S10 of the right lung, as well as an enlarged right hilar lymph node (Figure 2). Abdominal CT revealed no lesions in the liver, gallbladder, or pancreas. FDG-PET revealed uptake in the mass (SUV: 8.1) in the right lung and in the swelling of #10 lymph node (SUV: 4.1). No abnormal FDG accumulation was noted in any other organ. Serum biochemical tests did not reveal any evidence of hepatic dysfunction or hepatitis B or C. Of the tumor markers, serum carbohydrate 12-5 (CA12-5), neuron-specific enolase (NSE), Sialyl Lewis X (SLX), β-human chorionic gonadotropin (βHCG), pro-gastrin releasing peptide (PRO-GRF), and cytokeratin 19 fragment (CYFRA) levels were within normal range, while the serum AFP was markedly elevated (4620 ng/ml), and serum carcinoembryonic antigen (CEA; 6.6 ng/ml) and carbohydrate antigen 19-9 (CA19-9; 46.6 ng/ml) were slightly elevated. Transbronchial lung biopsy led to the diagnosis of AFP-producing lung carcinoma. Surgical treatment was selected on the basis of the preoperative tumor stage (T2N0M0). Right lower lobectomy + mediastinal lymph node dissection excision was carried out by video assisted thoracic surgery. The resected tumor measured 6.5 cm in diameter and was a solid tumor (Figure 3). Histopathologically, the tumor was composed of tumor cells with relatively irregular nuclear sizes and cylindrical, partially eosinophilic and dimly bright cell bodies showing little polymorphism, leading to the diagnosis of moderately differentiated adenocarcinoma (p0, pm0, ly1, v0). Lymph node metastasis was noted in #7,#10 and #11i lymph nodes, which led to a revision of the tumor stage to T2bN2M0/stageⅢA. Immnohistochemical staining for AFP revealed positive staining of a number of tumor cells for AFP, leading to the diagnosis of AFP-producing lung adenocarcinoma (Figure 4,5). The cells also showed positive staining for cytokeratine (CK)18, CK19, and anti-hepatocyte antibody. Thus, although the histological and morphological features of the tumor differed from those of hepatocellular carcinoma, the chromatic responses of the tumor to immunostaining were close to those known for hepatocytes. Of the indicators of the tumor malignancy grade, tumor protein 53 was negative, and the MIB-1 index was slightly high (40%). The postoperative course was favorable, and the serum AFP level returned to normal range by about 2 weeks after the surgery. At present, the patient is receiving adjuvant chemotherapy (Tegafur-Uracil), and has not, until date, shown any signs of tumor recurrence.

## Conclusions

AFP is one of the fetal proteins with a molecular weight that is intermediate between that of albumin and α1-globulin. It is produced by the fetal liver, yolk sac, and gastrointestinal cells. In relation to its pathological significance, serum AFP is useful as a tumor marker in patients with liver cancer. In adults showing elevated serum AFP levels, the malignant diseases requiring differential diagnosis include liver cancer, germ cell tumors (e.g., yolk sac tumor), and metastatic lung cancer, and the benign diseases requiring differential diagnosis include acute or chronic hepatitis, liver cirrhosis, and congenital biliary atresia [1-3]. It has been reported that AFP-producing tumors account for about 2%-8% of all cases of gastric cancer, and that the percentage is higher among cases of advanced gastric cancer [4,5]. Only a small number of reports have been published of cases with AFP-producing lung cancer; therefore, the pathophysiology and clinical characteristics of AFP-producing lung cancer have not yet been adequately clarified.

AFP-producing lung cancer was first reported by Corlin et al. [6] and has since been reported to account for about 2% of all lung cancers [7]. Histologically, adenocarcinoma (often poorly differentiated adenocarcinoma) accounts for the most of AFP-producing lung cancers. Furthermore, large-cell carcinoma accounts for 25% of all AFP-producing lung cancers. Thus, adenocarcinoma and large-cell carcinoma account for nearly all cases of AFP-producing lung cancer [8], although rare cases of AFP-producing squamous cell carcinoma [9] and AFP-producing carcinoid [10] have also been reported. As stated above, AFP-producing gastric cancer shows a high propensity for metastasizing to the liver and lymph nodes, and its prognosis is reported to be poorer as compared with that of non-AFP-producing gastric cancer. In relation to AFP-producing lung cancer, it must be kept in mind during the follow-up of these patients that the percentage of cases with poorly differentiated adenocarcinoma and the frequency of a high MIB-1 index are significantly higher in these cases than in those with non-AFP-producing liver cancer [11] during the follow-up of patients.

In regard to tumor markers, cases of AFP-producing liver cancer presenting with elevated serum CEA or HCG levels have been reported [12]. In the present case also, slight elevation of the serum CEA and CA19-9 levels was noted in addition to elevation of the serum AFP. The serum levels of these tumor markers returned to normal soon after tumor resection, and their pathological significance remained unknown.

The concept "hepatoid carcinoma" has been proposed in connection with this disease [13,14]. This concept is used to indicate adenocarcinoma composed of a mixture of hepatoid components (cancer assuming the form of a hepatocellular carcinoma) and papillary components. Cases of hepatoid carcinoma affecting the pancreas, kidney, duodenum, gallbladder, etc. have been reported. Immunohistochemically, AFP is found in both the hepatoid component and the papillary component of hepatoid carcinomas. If the hepatoid component is dominant, the term "hepatoid-adenocarcinoma" is used. When the tumor keratin expression profile was analyzed in the present case, a very small percentage of the cells were found to be positive for CK7, whereas CK20 and TIF-1 were negative, the profile thus differing slightly from the profile known for typical lung cancer. However, the tumor in our patient also differed from hepatocellular carcinoma in terms of the histological and morphological features, which eventually led to the final diagnosis of AFP-producing lung adenocarcinoma. The number of cases with this type of tumor reported until date is rather small. Further accumulation of cases and analysis of data on the malignancy grade and long-term prognosis of AFP-producing lung adenocarcinoma would be desirable.

## Consent statement

Informed consent was obtained from the patient for publication of this case report and accompanying images. A copy of the written consent is available for review by the Editor-in-Chief of this journal.

## Competing interests

The authors declare that they have no competing interests.

## Authors' contributions

MK have operated this case and analyzed all data. KO, and KS, YM, SH did the assistant of the operation. YT and NM diagnosed h the pathology of this case. TS was the professor of the surgical science and had a guide. All authors read and approved the final manuscript.

## References

[B1] Bergstrand CGQ Czar B Raper electrophoresis study of human total serum protein with demonstration of a new protein fraction Scand j Clin lab Invest 1957 9 277 286 10.3109/00365515709079971 13495347

[B2] Gitlin D Perricelli A Gitlin GM Synthesis of arpha-fetoprotein by liver yolk sac and Gastrointestinal tract of human conceptus Cancer Res 1972 32 979 982 4111729

[B3] Etarinov YS Content of embryo-apecific alpha-globlin in fetal and neonatal sera and sera from adult humans with primary carcinoma of the liver Fed Proc Transl Suppl 1966 25 344 346 4160512

[B4] Adachi Y Tsuchihashi J Shiraishi N Yasuda K Etoh T Kitano S AEP-producing gastric carcinomamultivariate analysis of prognostic factors in 270 patients Oncology 2003 21 357 362 10.1159/000072332 12931013

[B5] Chang YC Nagasue N Kohno H Taniura H Uchida M Yamanoi A Kimoto T Nakamura T Clinicopathologic features and long-term results of arufa-fetoprotein-producing gastric cancer Am J Gastroenterol 1990 85 1480 1485 1700600

[B6] Corlin RF Tompkins RK Serum alpha-fetoglobulin in a patient with hepatic metastasis from brobchogenic carcinoma Am J Dig Dis 1972 17 533 535 10.1007/BF02231210 4113134

[B7] Walop W Chrétien M Colman NC Fraser RS Gilbert F Hidvegi RS Hutchinson T Kelly B Lis M Spitzer WO The use of biomarkers in the prediction of survival in patients with pulmonary carcinoma Cancer 1990 65 2033 2046 10.1002/1097-0142(19900501)65:9<2033::AID-CNCR2820650925>3.0.CO;2-K 2164876

[B8] Okunaka T Kato H Konaka C Yamamoto H Furukawa K Primary Lung Cancer Producing Alpha-Fetoprotein Ann Thorac Surg 1992 53 151 152 10.1016/0003-4975(92)90778-3 1370196

[B9] Asamura H Nakayama H Kondo H Tsuchiya R Ono R Noguchi M Yoda H Naruke T AFP-producing squamous cell carcinoma of the lung in an adolescent Jpn J Clin Oncol 1996 26 103 106 8609692 10.1093/oxfordjournals.jjco.a023181

[B10] Yamagata T Yamagata Y Nakanishi M Matsunaga K Minakata Y Ichinose M A case of primary lung cancer producing alpha fetoprotein Can Respir J 2004 11 7 504 506 15505704 10.1155/2004/510350

[B11] Hiroshima K Iyoda A Toyozaki T Haga Y Baba M Fujisawa T Ishikura H Ohwada H Alpha-fetoprotein-producing lung carcinoma: Report of three cases Pathology International 2002 52 46 53 10.1046/j.1440-1827.2002.01311.x 11940206

[B12] Yoshino I Hayashi I Yano T Takai E Mizutani K Ichinose Y Alpha fetoprotein-producing adenocarcinoma of the lung Lung Cancer 1996 15 125 131 10.1016/0169-5002(96)00577-6 8865130

[B13] Arnould L Drouot F Fargeot P Bernard A Foucher P Collin F Petrella T Hepatoid Adenocarcinoma of the lung Report of a case of an unusual alpha fetoprotein-producing Lung Tumor Am J Surg Pathol1 1997 21 1113 1118 10.1097/00000478-199709000-00018 9298890

[B14] Hayashi Y Takanashi Y Ohsawa H Ishii H Nakatani Y Hepatoid adenocarcinoma in the lung. Lung Cancer 2002 38 211 4 10.1016/s0169-5002(02)00214-3 12399135

